# The E3 Ubiquitin Ligase SIAH1 Targets MyD88 for Proteasomal Degradation During Dengue Virus Infection

**DOI:** 10.3389/fmicb.2020.00024

**Published:** 2020-02-14

**Authors:** Ashleigh R. Murphy Schafer, Jessica L. Smith, Kara M. Pryke, Victor R. DeFilippis, Alec J. Hirsch

**Affiliations:** Vaccine and Gene Therapy Institute, Oregon Health & Science University, Beaverton, OR, United States

**Keywords:** dengue, flavivirus, unfolded protein response, microRNA, ubiquitin

## Abstract

The dengue virus presents a serious threat to human health globally and can cause severe, even life-threatening, illness. Dengue virus (DENV) is endemic on all continents except Antarctica, and it is estimated that more than 100 million people are infected each year. Herein, we further mine the data from a previously described screen for microRNAs (miRNAs) that block flavivirus replication. We use miR-424, a member of the miR-15/16 family, as a tool to further dissect the role of host cell proteins during DENV infection. We observed that miR-424 suppresses expression of the E3 ubiquitin ligase SIAH1, which is normally induced during dengue virus 2 (DENV2) infection through activation of the unfolded protein response (UPR). Specific siRNA-mediated knockdown of SIAH1 also results in inhibition of DENV replication, demonstrating that this target is at least partly responsible for the antiviral activity of miR-424. We further show that SIAH1 binds to and ubiquitinates the innate immune adaptor protein MyD88 and that the antiviral effect of SIAH1 knockdown is reduced in cells in which MyD88 has been deleted by CRISPR/Cas9 gene editing. Additionally, MyD88-dependent signaling, triggered either by DENV2 infection or the Toll-like receptor 7 (TLR7) ligand imiquimod, is increased in cells in which SIAH1 has been knocked down by miR-424 or a SIAH1-specific siRNA. These observations suggest an additional pathway by which DENV2 harnesses aspects of the UPR to dampen the host innate immune response and promote viral replication.

## Introduction

The dengue viruses (DENVs) consist of four antigenically distinct serotypes (DENV1–4) that together present considerable risk of infection in tropical and subtropical regions worldwide (Bhatt et al., 2013). DENV is a positive-stranded RNA genome flavivirus, primarily transmitted to humans by the bite of an infected *Aedes aegypti* mosquito. Infection with any of the DENV serotypes can result in a self-limiting febrile illness called dengue fever, often characterized by severe myalgia and a distinctive cutaneous rash. Life-threatening severe dengue illness, often associated with secondary infection by a heterologous DENV serotype, is characterized by thrombocytopenia, vascular leakage, and hypotensive shock. DENV-related disease results in millions of hospitalizations and thousands of deaths each year (Bhatt et al., 2013; Stanaway et al., 2016). Currently, antiviral therapeutics are not available for DENV infection, and the vaccine currently licensed in some countries offers less than optimal protection (Halstead, 2017).

Infection with DENV induces an antiviral immune state in the host cell through various pattern recognition receptors (PRRs) including Toll-like receptor (TLR)3, TLR7, retinoic acid–inducible gene I (RIG-I), and stimulator of interferon genes (STING) (Loo et al., 2008; Tsai et al., 2009; Sariol et al., 2011; Aguirre et al., 2012; Green et al., 2014). Binding of pathogen-associated molecular patterns (PAMPs), such as double-stranded RNA, to a PRR triggers a signaling cascade resulting in transcription of a multitude of infection response genes. DENV, like most viruses, employs a variety of direct and indirect methods to avoid, reduce, or disrupt antiviral immune signaling within the infected cell. The DENV non-structural protein NS5 binds directly to the interferon-stimulated cellular transcription factor signal transducer and activator of transcription (STAT)2, leading to the proteasomal degradation of STAT2 and a reduction in type 1 interferon signaling (Ashour et al., 2009; Morrison et al., 2013). Other DENV proteins also target immune signaling pathways: the DENV NS2B/3 protease complex cleaves STING, and NS4A and NS4B inhibit phosphorylation of STAT1 (Muñoz-Jordán et al., 2005; Rodriguez-Madoz et al., 2010; Aguirre et al., 2012). DENV also indirectly avoids detection by coopting structures and pathways of the cell. Infection by DENV results in extensive restructuring of the endoplasmic reticulum (ER), leading to the development of invaginations of the ER membrane, which may allow viral replication to occur without detection by dsRNA-sensing PRRs (Welsch et al., 2009; Rodriguez-Madoz et al., 2010; Pena and Harris, 2012). DENV infection also triggers portions of the unfolded protein response (UPR), mitigating proapoptotic stress signaling and ensuring continuous phospholipid and protein production (Pena et al., 2011).

MicroRNAs (miRNAs) are short, non-coding RNA segments that, in coordination with an RNA-binding protein complex, regulate translation of gene transcripts in a sequence-specific manner, based on partial complementarity between the miRNA and the 3′ UTR of the target mRNA (Agarwal et al., 2015). miRNAs have been proven to be useful tools for identifying interactions between viruses and the host cell (Santhakumar et al., 2010; Smith et al., 2012, 2017). We have previously identified miRNAs that inhibit replication of multiple flaviviruses using a high-content screen of human miRNAs (Smith et al., 2017). By examining the gene transcripts modulated by individual miRNAs or miRNA families with antiviral activity, we have identified cellular proteins and pathways required for flavivirus infection, including a previously undescribed role for WNT signaling in the interferon response to viral infection (Smith et al., 2017).

In the present study, we examine a family of antiviral miRNAs identified in our library screen, focusing in particular on miR-424, which has not previously been studied in the context of viral infection in spite of its potent anti-flaviviral effects. Many miRNAs target genes with similar functions (Finnerty et al., 2010; Meunier et al., 2013; Jiang et al., 2017). Herein, we observe that expression of miR-424 inhibits dengue virus 2 (DENV2) infection by repressing translation of three E3 ubiquitin ligases: SIAH1, SMURF1, and SMURF2. We show that DENV2 infection strongly induces SIAH1 expression, resulting in SIAH1 binding and ubiquitinating the TLR signaling adaptor protein, MyD88. Inhibition of SIAH1 expression, and thus preventing the subsequent proteasomal degradation of MyD88, results in significantly increased MyD88 protein expression as well as induction of genes dependent on TLR signaling through MyD88. These observations suggest an additional mechanism by which DENV2 disrupts TLR signaling activated cellular defenses through the induction of negative regulators of immune signaling.

## Materials and Methods

### Cell Culture and Virus Strains

HeLa (ATCC) cells were grown in Dulbecco modified Eagle medium (Thermo Fisher) supplemented with 5% fetal bovine serum (FBS; HyClone), 2 mM L-glutamine (Thermo Fisher), 100 U penicillin G sodium/ml, and 100 μg of streptomycin sulfate/ml. HEK293 (Microbix) cells were grown in modified Eagle medium (Thermo Fisher) supplemented with 5% FBS, 2 mM L-glutamine, 100 U penicillin G sodium/ml, and 100 μg of streptomycin sulfate/ml.

Dengue viruse 2 (New Guinea C) was obtained from ATCC. WNV (385-99) has been previously described (Xiao et al., 2001). DENV2 and WNV were passaged twice on *Aedes albopictus* mosquito–derived C6/36 cells and purified by centrifugation as previously described (Medigeshi et al., 2009). DENV2 and WNV virus titers were determined by focus-forming assay: virus was serially diluted and plated on Vero cells. Following 1 h incubation with rocking to allow adsorption, a 0.5% carboxymethyl cellulose (CMC; Sigma-Aldrich) overlay was added. The cells were fixed in 4% paraformaldehyde (PFA; Thermo Fisher) 48 h p.i., washed three times with phosphate-buffered saline (PBS), and then blocked and permeabilized in PBS containing 2% normal goat serum (NGS; Sigma-Aldrich) and 0.4% Triton X-100 (Thermo Fisher). The cells were then incubated for 2 h with 2 μg/ml anti-flavivirus envelope antibody 4G2 in PBS containing 2% NGS and 0.4% Triton X-100. Cells were then washed with PBS three more times and incubated with 0.4 μg/ml anti-mouse IgG-horseradish peroxidase secondary antibody (Santa Cruz Biotech) in PBS, containing 2% NGS, and 0.4% Triton X-100. Foci were visualized using the Vector VIP peroxidase substrate (Vector labs) as directed by the manufacturer.

Herpes simplex virus 1 (F1 strain) was a gift from A. Hill of Oregon Health and Science University. VacV (Western Reserve strain) was a gift from M. Slifka of Oregon Health and Science University. CHIKV (MH56 strain) was a gift of D. Streblow of Oregon Health and Science University. CHIKV, HSV, and VacV titers were determined by plaque-forming assay. Serial dilutions of virus were added to Vero cells, which were then incubated at 37° for 1 h and then overlayed with 0.5% CMC. Cells were fixed in 4%72 h p.i. Cells were stained with crystal violet.

Vesicular stomatitis virus-GFP (VSV) was a gift from V. DeFilippis of Oregon Health and Science University. VSV-GFP titers were determined by fluorescent plaque: 24 h p.i. with serially diluted virus, Vero cells were fixed in 4% PFA and plaques examined using fluorescent microscopy.

In all infections, cells were incubated for 1 h with virus at the indicated MOI in a low volume of medium containing 2% FBS, washed with PBS, and refed with medium containing 2% FBS, 2 mM L-glutamine, 100 U penicillin G sodium/ml, and 100 μg of streptomycin sulfate/ml.

### Reagents

Where indicated, cells were incubated with 1 μM thapsigargin (Sigma-Aldrich) in complete medium for 6 h unless otherwise noted. Cells treated with MG132 (Thermo Fisher) were incubated for 6 h in medium containing 5 μM MG132 for 6 h unless otherwise noted. In experiments employing imiquimod treatment, cells were incubated with 1 μg/ml imiquimod (Enzo Life Sciences) for 24 h in complete medium. The antibodies used were: anti-MyD88 (Cell Signaling Technology), anti-myc tag (Life Technology), anti-FLAG tag (Sigma-Aldrich), anti-GAPDH (Abcam), and anti-flavivirus E antibody 4G2 [hybridoma purchased from ATCC and maintained by the Vaccine and Gene Therapy Institute monoclonal antibody core facility (OHSU)].

### Plasmids

The 3′ UTRs of SIAH1, SMURF1, and SMURF2 were amplified by PCR (SIAH1-A, 5′-CTCGAGCACCCATCTGTCTGCCAA CC-3′; SIAH1-B, 5′-GCGGCCGCCTGTGCATGACGATGCCTT CTTC-3′; SMURF1-1-A, 5′-CTCGAGGCTGCTCTCCAATGCC ATCAG-3′; SMURF1-1-B, 5′-GCGGCCGCCGTAGCCTTCGG GCAGTTC-3′; SMURF1-2-A, 5′-CTCGAGGTTCTACTTTGGG TCCGCG-3′; SMURF1-2-B, 5′-GCGGCCGCCCAGCCTGCTG TACAATCAC-3′; SMURF2-A, 5′-TTGCGGCCGCCCAGCCTG CTGTACAATCAC-3′; SMURF2-B, 5′-TTGCGGCCGCGAGAT GGGTCTGCAACCAG-3′). PCR products were digested with the restriction enzymes *Not*I and *Xho*I (New England Biolabs) and ligated into reporter plasmid psiCHECK2 (Promega). Full-length SIAH1 and MyD88 were amplified by PCR (SIAH1-A, 5′-GCGGCCGCCCACCATGAGCCGTCAGAC-3′; SIAH1-B, 5′-CTCGAGACACATGGAAATAGTTACATTGATGCC-3′; My D88-A, 5′- GGATCCCCACCATGCGACCCGACCG-3′; MyD 88-B, 5′- GCGGCCGCTGGGCAGGGACAAGGCCTTGGC-3′). PCR products were digested with *Not*I and XhoIand ligated into pcDNA4 (Thermo Fisher) or pEF1/myc-His B (Thermo Fisher). The Ub-myc plasmid was a kind gift from A. Moses of Oregon Health and Science University.

### miRNA, siRNA, and Plasmid Transfections

miRIDIAN miRNA mimics (miR-424, C-300717-05; miR-15a, C-300482-03; miR-15b, C-300587-05; miR-16, C-300484-05; miR-103, C-301298-00; miR-107, C-300527-03; miR-195, C-300643-03; miR-497, C-300643-03; miR-503, C-300841-05; miR-646, C-300973-01; miR-199a-3p, C-300535-05; RISC-free negative control, D-001220-01) were purchased from Dharmacon. siRNAs targeting cellular mRNA (SIAH1, 142624; SMURF1, 120615; SMURF2, 120654) were purchased from Thermo Fisher. The DENV2 siRNA (5′- UGCUGAAACGCGAGAGAAA-3′) has been previously described (Stein et al., 2011). miRNA mimics and siRNAs were transfected at a concentration of 0.4 nM, using Lipofectamine RNAiMAX reagents (Thermo Fisher) according to manufacturer protocols. Plasmids expressing SIAH1, SMURF1, and SMURF2 cDNA or 3′ UTRs were transfected using Lipofectamine 3000 (Thermo Fisher) according to manufacturer recommendations. Cotransfections of plasmids and miRNA duplexes or siRNAs were also performed with Lipofectamine 3000.

### Lentivector Transduction and CRISPR/Cas9-Mediated Genome Editing

Genome editing using lentivector-mediated delivery of CRISPR/Cas9 components was performed as described previously (Sanjana et al., 2014; Pryke et al., 2017). Briefly, a 20-nucleotide guide RNA (gRNA) sequence targeting the MyD88 protein-coding region (5′-GCTCCAGCAGCACGTCGTCG-3′) was inserted into the lentiCRISPRv2 vector (AddGene 52961). Lentivirus was made by transfecting lentiCRISPRv2 plasmid along with packaging plasmid (psPAX2; AddGene 12260) and VSV G protein pseudotyping plasmid (pMD2.G; Addgene 12259) into Lenti-X 293T cells (Clontech) using Lipofectamine-LTX (Life Technologies, Inc.). Medium was harvested at 48 and 72 h post-transfection, centrifuged (3,000 × *g* for 10 min), and filtered through a 0.45 μm filter to remove cell debris. Subconfluent HeLa cells were exposed to lentivirus for 8 h in the presence of 5 μg/ml Polybrene. After the cells reached confluence, they were split into DMEM plus 10% FBS containing 3 μg/ml hygromycin. Transduced cells were passaged in the presence of hygromycin for 7–10 days before protein knockout was examined by immunoblotting.

### Immunofluorescence

Infected cells were fixed in 4% PFA and washed two times with a wash buffer consisting of PBS containing 0.2% BSA and 0.2% Triton X-100, and then incubated for 1 h in blocking buffer composed of 2% BSA and 0.2% Triton X-100 in PBS. Cells were next incubated in 2 μg 4G2 antibody/ml wash buffer for 1 h, washed three times with additional wash buffer, and incubated 1 h with 1 μg goat α-mouse Alexa Fluor 488 (Thermo Fisher)/ml wash buffer. Cells were washed several times, with one wash containing 4′,6-diamidino-2-phenylindole (DAPI; Thermo Fisher) to stain cell nuclei, and visualized by fluorescent microscopy.

### Luciferase Assays

HEK293 cells were transfected with psiCHECK2 reporter plasmids and either the miR-424 mimic or negative control mimic using Lipofectamine 3000 (Thermo Fisher) according to manufacturer recommendations. *Renilla* (test) and firefly (control) luciferase activity was measured 48 h post-transfection, using the Dual Luciferase Reporter Assay System (Promega) according to the manufacturer’s protocol.

### qPCR

100 ng of total RNA was reverse-transcribed and amplified with SIAH1 (Hs02339360_m1)–, SMURF1 (Hs00410929_m1)–, SMURF2 (Hs00224203_m1)–, IL-6 (Hs00985639_m1)–, IL-8 (Hs00174103_m1)–, IFIT1 (Hs01911452_s1)–, IFIT2 (Hs00533665_m1)–, or beta-actin (Hs99999903_m1)–specific Taqman gene expression probes (Thermo Fisher) and Taqman RNA-to-CT 1-Step reagents (Applied Biosystems) on a StepOne Plus real-time PCR instrument (Applied Biosystems) based on the manufacturer’s recommendations. Relative transcript expression of the genes of interest was calculated using the ΔΔCt method (Schmittgen and Livak, 2008) with beta-actin serving as the endogenous control transcript.

### General Statistical Analysis

Experiments were performed at least three times unless otherwise indicated in the figure legends. Data are presented as mean ± standard error of the mean (SEM). Student *t* test was used for single time point viral titers and luciferase assays when two sets were compared. For data containing more than two sets or more than a single time point, a one-way ANOVA analysis was conducted with Bonferroni’s correction for *post hoc* multiple comparisons. A *p*-value < 0.05 was considered significant.

## Results

### miR-15/16 miRNA Family Members Inhibit Flavivirus Replication

**FIGURE 1:**
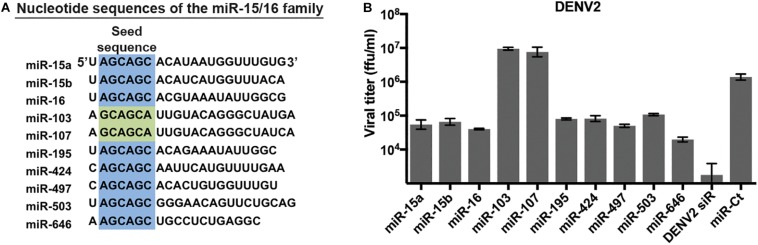
MicroRNAs (miRNAs) of the miR-15/16 family with identical seed sequences inhibit dengue virus 2 (DENV2). **(A)** Comparison of the seed sequences of the members of the miR-15/16 family of miRNAs. Identical seed sequences (nucleotides 2–7) are highlighted in blue; the seed sequences of miR-103 and miR-107 (in green) are similar but offset by one nucleotide. **(B)** HeLa cells were transfected with the indicated miR-15/16 family member duplex RNA mimics, a validated non-targeting miRNA mimic (miR-Ct) as a negative control, or a DENV2 genome siRNA (DENV2 siR) and infected with DENV2 (MOI = 3 ffu/cell) 2 days post-transfection. Supernatants were collected 3 days p.i. and titered on Vero cells.

We have previously identified miRNAs that inhibit replication of multiple flaviviruses using a high-content screen of double-stranded miRNA mimics (Smith et al., 2017). Of note, several miRNA families – generally defined by similarity within the seed sequence – were found to have multiple members with anti-flavivirus activity in this assay. One such miRNA family is the miR-15/16 family, which consists of 10 miRNAs with similar nucleotide sequences (Finnerty et al., 2010). Eight of the 10 miRNAs have an identical 6mer sequence in positions 2–7 of the seed sequence (Figure 1A), while the remaining 2, miR-103 and miR-107, have the sequence offset by one nucleotide. We re-examined the ability of miR-15/16 family members to inhibit DENV replication. HeLa cells were transfected with the duplex mimic of miR-15/16 family members and infected with DENV2 (Figure 1B). Supernatants collected 3 days p.i. contained viral titers that were reduced 15- to 70-fold in cells transfected with miR-424, miR-15a, miR-15b, miR-16, miR-195, miR-497, miR-503, and miR-646, all of which share identical seed sequences. The remaining two members of the miR-15/16 family, miR-103, and miR-107, did not appear to inhibit DENV2 infection. While miR-103 and miR-107 are similar in sequence to the rest of the family, the miRNAs do not share the same seed sequence, demonstrating that the 5′-AGCAGC-3′ seed is crucial for the antiviral activity of this miRNA family.

**FIGURE 2:**
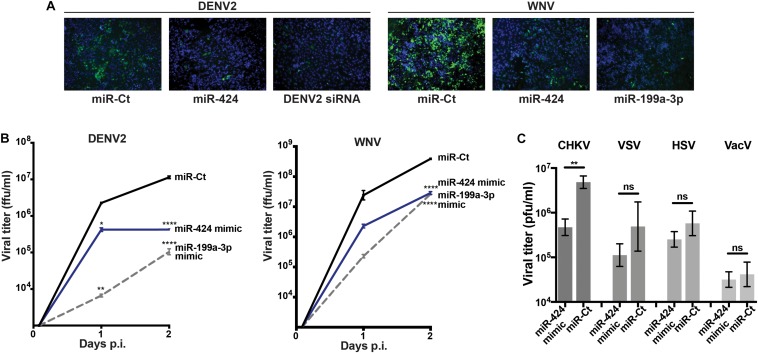
Expression of a miR-424 mimic inhibits RNA virus infection. **(A)** HeLa cells were transfected with a duplex RNA mimic of miR-424, the broadly antiviral miR-199a-3p, an siRNA against the DENV2 genome (DENV2 siRNA), or a negative control miRNA mimic (miR-Ct). Two days post-transfection, cells were infected with DENV2 (MOI = 10 ffu/cell) or WNV (MOI = 3 ffu/cell). Two (WNV) or 3 (DENV2) days p.i., cells were fixed and incubated with anti-flavivirus E antibody, and infected cells visualized with an Alexa Fluor–conjugated anti-mouse immunoglobulin antibody (green). Cell nuclei were stained with 4′,6-diamidino-2-phenylindole (DAPI) (blue). **(B)** Supernatants were collected from the above infected cells at 2 h, 1 day, and 2 days p.i., and infectious viral titers [displayed as focus-forming unit (ffu)/ml] determined by focus-forming assay on Vero cells. **(C)** HeLa cells transfected with the miR-424 mimic or negative control miRNA mimic were infected with CHKV, vesicular stomatitis virus (VSV), herpes simplex virus (HSV), or vaccinia virus (VacV) (MOI = 0.5 pfu/cell). Supernatants were collected 3 days p.i., and titers [displayed as plaque-forming unit (pfu)/ml] determined by plaque-forming assay on Vero cells. Results are representative of at least three independent experiments. (* *p*-value < 0.05, ** *p*-value < 0.01, **** *p*-value < 0.001).

We next focused on miR-424, a member of the miR-15/16 family that had not previously been studied during RNA virus infection, in order to elucidate its activity against additional flaviviruses as well as other virus families. HeLa cells were transfected with a double-stranded RNA mimic of miR-424, infected with DENV2 or WNV and fixed at 3 (DENV2) or 2 (WNV) days post-transfection. Expression of the flavivirus E protein was detected by indirect immunofluorescence. A DENV2 genome-specific siRNA or the broadly antiviral miRNA miR-199a-3p was used as positive control for viral inhibition (Santhakumar et al., 2010). Cells transfected with the miR-424 mimic showed inhibition of E protein expression (Figure 2A). When supernatants were collected and virus quantified by focus-forming assay, titers from cells transfected with the miR-424 mimic were at least 10-fold lower than control cells when infected with DENV2 or WNV (Figure 2B). Similarly, miR-424 reduced the infectious titer produced when cells were infected with Chikungunya virus (CHIKV), a member of the positive-strand RNA alphavirus genus (Figure 2C). In contrast, miR-424 did not impair infection by a negative-stranded RNA virus (VSV) or by the DNA viruses, herpes simplex virus (HSV), and vaccinia virus (VacV).

### miR-424 Targets Several Cellular E3 Ubiquitin Ligases

**FIGURE 3:**
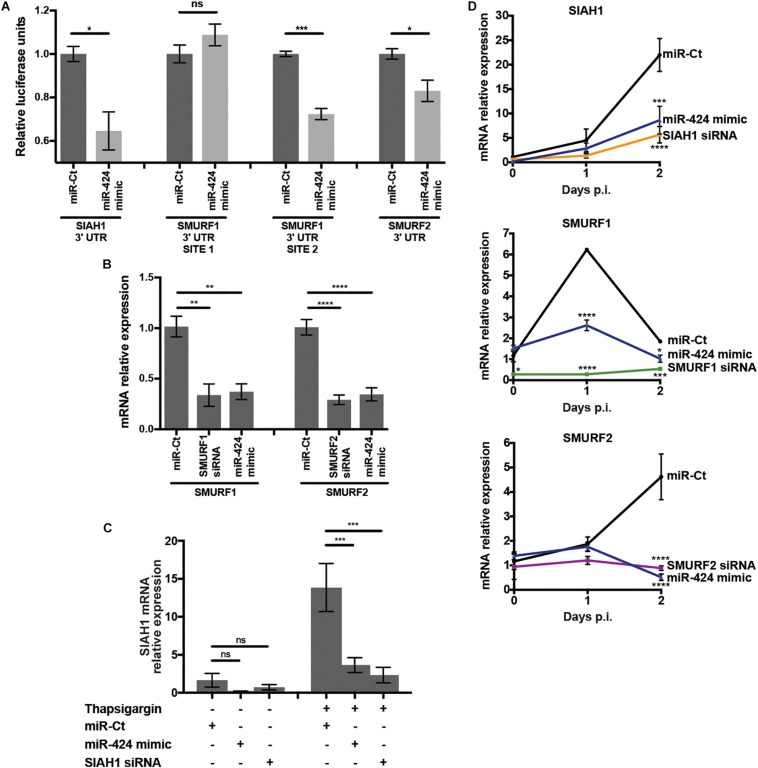
miR-424 targets the 3′ UTR of SIAH1, SMURF1, and SMURF2, inhibiting transcript expression in uninfected and DENV2 infected cells. **(A)** Dual luciferase reporter plasmids were constructed containing the 3′ UTRs of SIAH1, SMURF1, or SMURF2. The 3′ UTR of SMURF1 contains two putative miR-424 binding sites, prompting the construction of two plasmids containing one binding site each. HEK293 cells were transfected with the reporter plasmid and the miR-424 mimic or control small RNA. Cells were lysed 48 h post-transfection, and luciferase expression measured by luminometer. Activity is represented as relative luciferase units (RLUs), calculated as test normalized to control. **(B)** HeLa cells were transfected with siRNA against SMURF1 or SMURF2, the miR-424 mimic, or a control mimic, and 48 h post-transfection total RNA was collected in Trizol. Relative expression of the SMURF1 and SMURF2 mRNAs was determined by qPCR and normalized to β-actin. **(C)** HeLa cells were transfected with the SIAH1 siRNA, the miR-424 mimic, or control mimic. At 48 h post-transfection, they were treated with thapsigargin (1 μM) for 6 h. Total RNA in Trizol was collected and relative mRNA levels of SIAH1 determined as described above. **(D)** HeLa cells were transfected as indicated and infected with DENV2 (MOI = 5 ffu/cell). Total RNA was collected in Trizol at the indicated time points and relative mRNA levels determined as described above. (* *p*-value < 0.05, ** *p*-value < 0.01, *** *p*-value < 0.005, **** *p*-value < 0.001).

miR-424 target prediction via *in silico* analysis (Agarwal et al., 2015) and experimental evidence (Dallavalle et al., 2016) indicates that genes related to ubiquitin conjugation were highly represented, suggesting a role for miR-424 in regulation of protein stability via control of ubiquitin. In particular, E3 ubiquitin ligases were frequently predicted targets of miR-424 (Agarwal et al., 2015). Notably, the ubiquitin-proteasome system (UPS) is essential for DENV and WNV infection, and inhibition of the UPS severely impairs replication (Fernandez-Garcia et al., 2011; Padilla-S et al., 2014; Byk et al., 2016). Therefore, we hypothesized that perturbation of the UPS via miR-424–mediated inhibition of expression of one or more E3 ubiquitin ligases may contribute to inhibition of DENV and other flaviviruses. We initially examined repression of the E3 ubiquitin ligases SIAH1, SMURF1, and SMURF2, which have been identified as targets of miR-424 in several cancers as well as pulmonary fibrosis (Imig et al., 2011; Wu et al., 2013; Xiao et al., 2015). To verify modulation of SIAH1, SMURF1, and SMURF2 by miR-424 in our system, the 3′ UTR of each gene, containing the predicted binding site of miR-424, was cloned into the dual luciferase reporter system psiCHECK-2. SMURF1 contains two sites complementary to the miR-424 seed sequence, which were each cloned into separate plasmids. Cells were co-transfected with the reporter plasmid and the miR-424 mimic, and luciferase activity measured in cell lysates (Figure 3A). Reporter expression was reduced 35% or 15% when miR-424 was present in cells cotransfected with the SIAH1 or SMURF2 3′ UTRs (respectively). Of the two putative miR-424 binding sites in the SMURF1 3′ UTR, only the downstream site resulted in reduced luciferase expression when miR-424 was present, consistent with the binding site identified by Xiao et al. (2015).

To examine the effect of the miR-424 mimic on expression of the endogenous mRNA transcripts of SIAH1, SMURF1, and SMURF2, RNA was collected from HeLa cells transfected with the miR-424 mimic and transcript expression measured by qPCR. The miR-424 mimic reduced the constitutive transcript expression of both SMURF1 and SMURF2 to a similar extent as siRNAs specific for those transcripts (Figure 3B). In contrast to SMURF1 and 2, SIAH1 is expressed at very low constitutive levels; however, SIAH1 expression can be induced by the UPR (Scortegagna et al., 2014). As expected, we detected very low levels of SIAH1 mRNA in untreated HeLa cells but much higher levels in cells treated with thapsigargin, which inhibits calcium exchange in the ER, causing ER stress and inducing the UPR (Figure 3C). In cells transfected with the miR-424 mimic and subsequently treated with thapsigargin, SIAH1 expression was significantly reduced.

Flavivirus infection has been shown to activate several arms of the UPR, suggesting that SIAH1 expression may be induced in cells infected with DENV2 (Medigeshi et al., 2007; Pena et al., 2011; Pena and Harris, 2012; Scortegagna et al., 2014). RNA was collected from HeLa cells 24 and 48 h after DENV2 infection and qPCR used to determine SIAH1 transcript levels (Figure 3D). SIAH1 mRNA was detectable at 24 h p.i. and increased to more than 20-fold above constitutive levels by 48 h post-infection. Cells transfected with the miR-424 mimic produced much less SIAH1 during DENV2 infection, indicating miR-424 inhibits SIAH1 mRNA expression during DENV2 infection. SMURF1 and SMURF2 transcripts were detectable prior to infection and showed moderate induction during DENV2 infection, although SMURF1 expression dropped to near constitutive levels by 48 h (Figure 3D). Like SIAH1, miR-424 inhibited expression of SMURF1 and SMURF2 during DENV2 infection.

### Knockdown of SIAH1 and SMURF2 E3 Ubiquitin Ligases Inhibits DENV2 Infection

**FIGURE 4:**
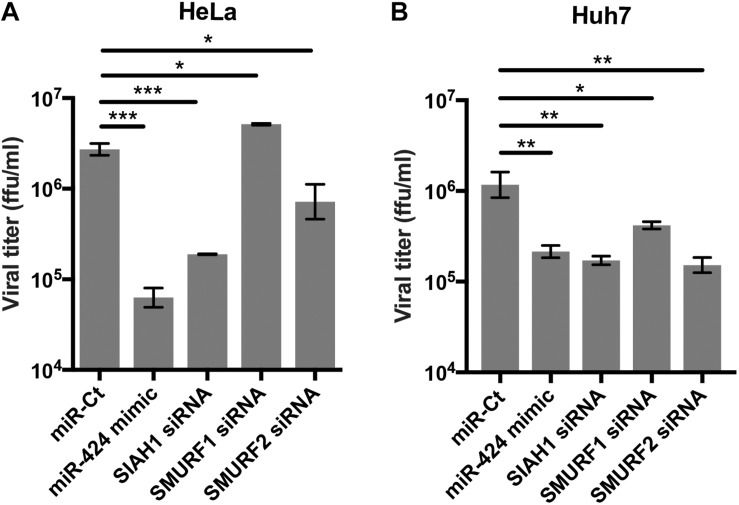
Inhibition of SIAH1 expression recapitulates the antiviral effect of miR-424 during DENV2 infection. HeLa cells **(A)** or Huh7 cells **(B)** were transfected as indicated and infected with DENV2 (MOI = 5 ffu/cell) 48 h post-transfection. Supernatants from infected cells were collected 48 h p.i. and titered on Vero cells. (* *p*-value < 0.05, ** *p*-value < 0.01, *** *p*-value < 0.005).

To determine if SIAH1, SMURF1, and SMURF2 are required for DENV2 infection, cells were transfected with siRNA against one of the three ligases prior to infection (Figure 4A). Knockdown of SIAH1 reduced viral titer released from infected cells at 48 h p.i. The extent to which DENV2 infection was inhibited by the SIAH1 siRNA was similar to the inhibition seen in miR-424 mimic transfected cells. Infection was also inhibited by SMURF2 knockdown, although to a lesser extent than the SIAH1 knockdown or miR-424 expression. In contrast, the SMURF1 siRNA did not inhibit DENV2 replication. These results suggest that repression of SIAH1 expression plays an important role in the antiviral effects of miR-424, although other genes regulated by miR-424 may also contribute to the overall inhibition of DENV2 infection. Because DENV-associated pathology is often associated with liver tropism of the virus, we also performed these experiments in Huh7 cells, a cell line derived from a hepatocellular carcinoma that supports DENV2 replication (Lin et al., 2000). As shown (Figure 4B), transfection of the miR-424 mimic as well as SIAH1, SMURF1, and SMURF2-specific siRNAs resulted in a decrease of viral titer compared to control transfected cells.

### SIAH1 Knockdown Inhibits Proteasome-Dependent Degradation of MyD88

**FIGURE 5:**
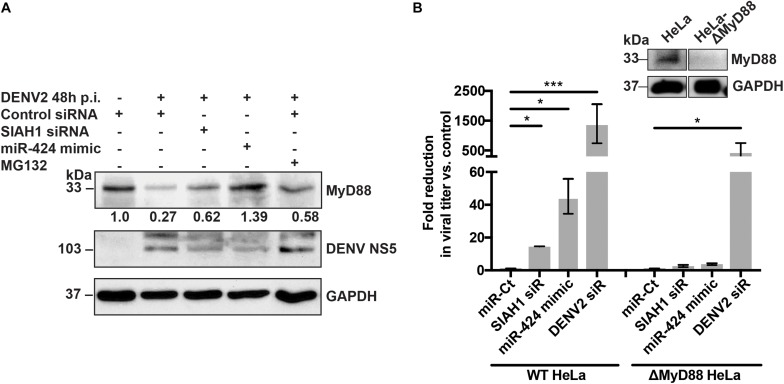
SIAH1 knockdown inhibits proteasome-dependent degradation of MyD88. **(A)** HeLa cells were transfected with as indicated and infected with DENV2 (MOI = 5 ffu/cell) 48 h post-transfection. At 42 h p.i., a cohort of siRNA control transfected wells were treated with MG132 to inhibit the proteasome. Cell lysates were collected 48 h p.i. and analyzed by western blot for MyD88 expression. **(B)** Wild-type (WT) and MyD88-deficient HeLa cells were transfected as indicated, infected with DENV2 (MOI = 5 ffu/ml) 48 h post-transfection. Supernatants were collected 48 h p.i. and titered on Vero cells. Fold reduction in viral titer represents the ratio of ffu/ml from control cells to transfected cells. MyD88 knockout was confirmed by western blot (inset). (* *p*-value < 0.05, *** *p*-value < 0.005).

Many of the miRNAs identified as inhibitors of flaviviruses act through modulation of cellular innate immune pathways (Smith et al., 2017 and unpublished data). Notably, previous studies have identified SIAH1 as a potential binding partner of the TLR signaling adaptor MyD88, suggesting that SIAH1 may play a role in regulating MyD88 through ubiquitination (Wang et al., 2011; De Arras et al., 2013). To determine if MyD88 protein expression is altered during DENV2 infection, cell lysates from infected cells were collected, subjected to SDS-PAGE, and MyD88 protein visualized by western blotting. At 48 h p.i., expression of MyD88 was reduced considerably as compared to uninfected cells (Figure 5A, lanes 1–2). However, in cells that were transfected with the SIAH1 siRNA or miR-424 mimic prior to infection, MyD88 expression was restored, suggesting that SIAH1 plays a role in the repression of MyD88 in DENV2 infected cells (Figure 5A, lanes 3–4). Inhibition of the proteasome using the drug MG132 also resulted in increased MyD88 expression, indicating that DENV2-mediated degradation of MyD88 is proteasome dependent (Figure 5A, lane 5).

To address the effect of SIAH1 inhibition on infected cells in the absence of MyD88 expression, we generated a HeLa cell line deficient in MyD88, using the CRISPR/Cas9 gene editing system (Sanjana et al., 2014; Sali et al., 2015). As expected, viral titers collected from cells with intact MyD88 were 14- or 44-fold reduced in cells transfected with the SIAH1 siRNA or miR-424 mimic (respectively) prior to infection, as compared to control transfected cells. In contrast, the strong reduction in viral titer was not observed in the MyD88-deficient cells (Figure 5B). These results suggest that the observed inhibition of DENV infection by miR-424 results from the repression of SIAH1-dependent degradation of MyD88.

### SIAH1 Binds and Ubiquitinates MyD88

**FIGURE 6:**
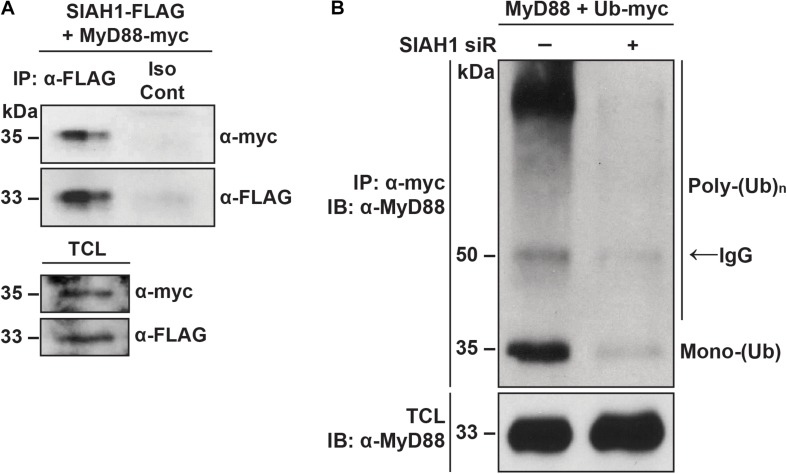
SIAH1 binds and ubiquitinates MyD88. **(A)** HeLa cells were treated with MG132 for 6 h and then transfected with plasmids expressing SIAH1-FLAG and MyD88-myc in the presence of MG132. Twenty-four hours post-transfection, total cell lysates were collected, immunoprecipitated with α-FLAG or an isotype control antibody, and analyzed by western blot (TCL, total cell lysate). **(B)** HeLa cells were transfected with or without SIAH1 siRNA as well as with plasmids expressing MyD88-FLAG and Ub-myc. Twenty-four hours post-transfection, cells were treated with thapsigargin to induce SIAH1 expression, and 2 days post-transfection with MG132 to inhibit the proteasome. Total cell lysates were immunoprecipitated with α-myc and analyzed by western blot.

Because we observed that degradation of MyD88 during infection occurs in a proteasome-dependent manner, we next examined the physical interaction between MyD88 and SIAH1. Cells were co-transfected with plasmids encoding MyD88 with a C-terminal myc epitope and SIAH1 with a C-terminal FLAG epitope tag. At 48 h post-transfection, cell lysates were collected and immunoprecipitated with a FLAG antibody or an isotype control. Western blotting revealed MyD88 in samples immunoprecipitated with FLAG but not in the control samples, indicating physical interaction between SIAH1 and MyD88 (Figure 6A).

We next examined the effect of SIAH1 on ubiquitination of MyD88. Cells were transfected with plasmids encoding MyD88 and myc-tagged ubiquitin. SIAH1 expression was modulated by co-transfection of a SIAH1 siRNA or negative control small RNA. At 24 h post-transfection, cells were treated with thapsigargin overnight to induce expression of SIAH1 via the UPR, followed by a 6 h treatment with MG132 to induce accumulation of ubiquitinated proteins. Cell lysates were collected and subjected to immunoprecipitation with an anti-myc antibody. Immunoprecipitated proteins were resolved by SDS-PAGE and MyD88 detected by western blotting with a MyD88-specific antibody. In control cell lysates, we detected mono- and poly-ubiquitinated MyD88 immunoprecipitated with the myc-tagged Ub (Figure 6B). In contrast, when SIAH1 expression was inhibited with the siRNA, very low levels of MyD88 ubiquitination were observed. These results suggest that SIAH1 binds MyD88 and directs its ubiquitination and support the hypothesis that the induction of SIAH1 during DENV infection decreases MyD88 expression.

### Inhibition of SIAH1 Increases MyD88-Mediated NF-κB Signaling During DENV2 Infection

**FIGURE 7:**
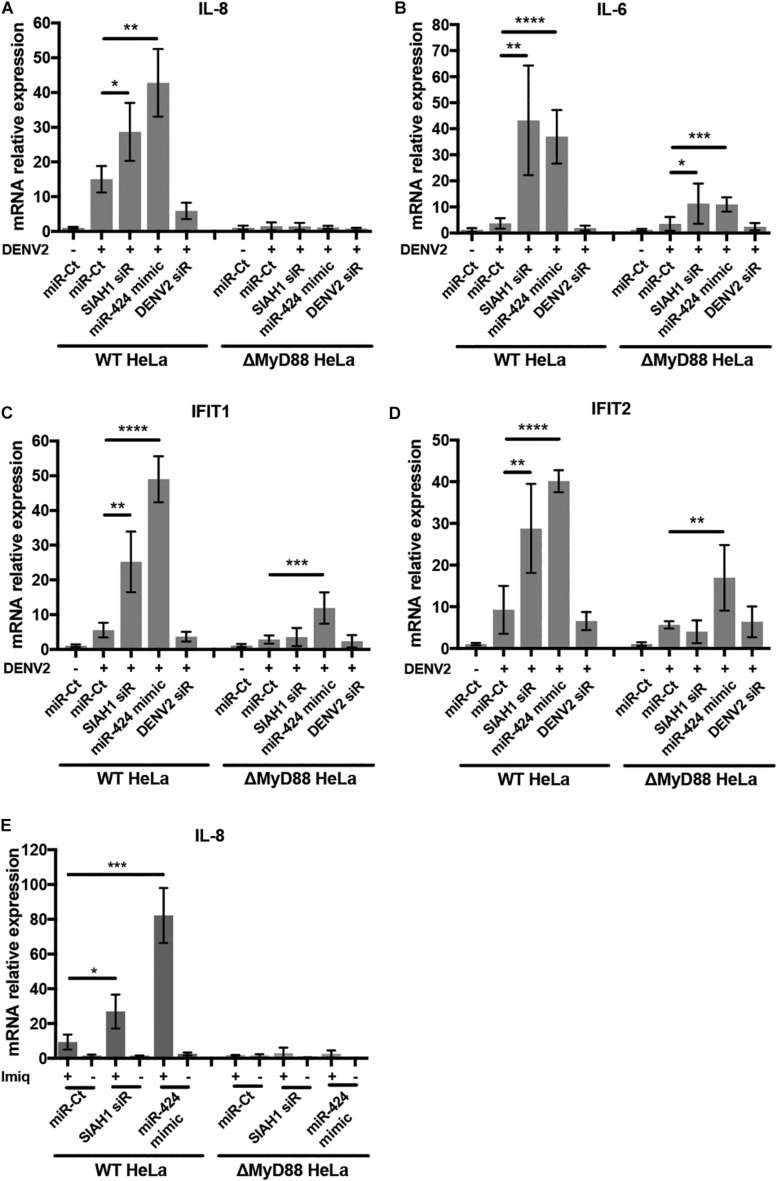
Inhibition of SIAH1 increases expression of NF-κB- and interferon-induced genes. **(A–D)** HeLa cells were transfected with the indicated siRNA or miRNA mimic and infected with DENV2 (MOI = 5 ffu/ml) 48 h post-transfection. Total RNA was collected in Trizol 48 h p.i., and relative transcripts levels determined by qPCR normalized to β-actin and uninfected control mRNA levels. **(E)** 48 h after transfection, cells were treated with imiquimod (10 μg/ml) for 24 h. Total RNA was collected and relative mRNA levels determined as described above. (* *p*-value < 0.05, ** *p*-value < 0.01, *** *p*-value < 0.005, **** *p*-value < 0.001).

MyD88 acts as an adaptor downstream of TLRs and the interleukin-1 receptor (IL-1R). Signal transduction through MyD88 activates of NF-κB and results in the upregulation of multiple innate immune factors, including interferon β and proinflammatory cytokines (Akira and Takeda, 2004). We therefore sought to determine if the antiviral effects of SIAH1 knockdown and miR-424 expression are a result of increased MyD88-dependent intracellular immune signaling during infection. We examined the induction of genes activated by NF-κB and interferon signaling during DENV2 infection in wild-type (WT) and MyD88-deficient HeLa cells. We used qPCR to measure the gene expression of the NF-κB–activated genes IL-8 and IL-6 as well as the interferon-stimulated genes (ISGs) IFIT1 and IFIT2 in MyD88-deficient cells and MyD88-intact cells during DENV2 infection. In cells with intact MyD88, expression of all four genes was increased after infection with DENV2 as expected. However, the induction of mRNA expression was substantially higher in cells transfected with the SIAH1 siRNA or the miR-424 mimic (Figures 7A–D). In contrast, when the MyD88-deficient cells were infected with DENV2, induction of mRNA levels was reduced compared to WT cells, and further induction of IL-8, IL-6, IFIT, and IFIT2 expression by SIAH1 siRNA was also absent. In the MyD88-deficient cells, the ability of miR-424 to enhance expression of these genes was also reduced.

To verify the role of SIAH1 in modulating MyD88-dependent signaling, we examined gene expression induced by activation of TLR7, which depends solely on MyD88 for signaling. WT and MyD88-deficient HeLa cells were transfected with the SIAH1 siRNA, the miR-424 mimic, or a control miR, followed by treatment with the drug imiquimod, a guanosine analog that specifically activates TLR7 (Figure 7E; Hemmi et al., 2002). Signaling through TLR7/MyD88 was assessed by induction of IL-8 mRNA using qPCR. As shown, imiquimod treatment increased IL-8 expression ∼10-fold in control transfected cells. In cells transfected with the SIAH1 siRNA or miR-424 mimic, IL-8 expression was substantially increased over the control transfected cells. In MyD88-deficient cells, no IL-8 induction was observed (Figure 7A). These data demonstrate that inhibition of SIAH1 expression results in increased NF-κB–regulated cytokine expression in a MyD88-dependent manner.

## Discussion

In a previously described high-content screen for cellular miRNAs capable of inhibiting flavivirus replication (Smith et al., 2017), we identified members of the miR-15/16 family as potent inhibitors of these viruses. In this study, we focused on a representative member of this family, miR-424, and determined that its antiviral effect is mediated through repression of the E3 ubiquitin ligase SIAH1. Expression of SIAH1 is induced by DENV2 infection, consistent with previous reports showing SIAH1 induction by the UPR (Scortegagna et al., 2014), and siRNA-mediated knockdown of SIAH1 inhibits DENV2 replication, similarly to miR-424 transfection. Furthermore, we show that SIAH1 binds and ubiquitinates MyD88 and that MyD88 is degraded in a proteasome-dependent manner during DENV2 infection. Inhibition of SIAH1 during infection results in increased innate immune signaling, but this increase, as well as inhibition of viral replication, is abrogated in cells in which the MyD88 gene has been deleted. These data demonstrate that SIAH1 acts as a proviral factor during DENV2 replication by contributing to attenuation of the host innate immune response.

Although the murine ortholog of miR-424, miR-322, is downregulated by activation of the UPR in mouse embryonic fibroblasts (Gupta et al., 2015), miR-424 is not constitutively expressed in the cells used in this study (Chamorro-Jorganes et al., 2011), nor did we detect the miRNA during DENV2 or WNV infection (data not shown). The absence of endogenous miR-424 expression allowed us to use the miR-424 mimic as a tool to identify cellular factors required for viral replication. Other constitutively expressed members of the miR-15/16 family may serve to limit UPR induction, preventing spurious activation and increasing rate of return to normal cellular homeostasis (Leung and Sharp, 2010), but additional studies will be required to examine the expression and function of the individual miR-15/16 family members. In this study, the exogenous expression of miR-424 significantly impaired infection and provided a finite pool of miR-424–binding targets from which to draw candidates for further study. Although we focused primarily on SIAH1 in this study, miR-424 targets many gene transcripts that may also contribute to the antiviral effect of the miRNA. For example, as we have shown here with SIAH1, inhibition of SMURF1 and SMURF2 expression resulted in increased MyD88 protein levels (data not shown), suggesting that total MyD88 degradation during DENV2 may be a cumulative result of these three E3 ubiquitin ligases. Other E3 ubiquitin ligase targets of miR-424 and the miR-15/16 family may also represent proviral factors during DENV2 infection.

Various studies have described the role of the UPR in DENV replication, though the observed effects of the UPR on replication vary, dependent on the virus, viral strain, and cell culture conditions (Yu et al., 2006; Pena et al., 2011; Diwaker et al., 2015). The UPR is made up of several signaling pathways activated in response to cellular stressors that disrupt protein translation and folding in the ER. Of the three arms of the UPR, the protein kinase RNA-like ER kinase (PERK) and activating transcript 6 (ATF6) pathways are active only transiently during DENV infection. In contrast, activation of the inositol-requiring protein-1 (IRE1) pathway appears about mid-way through DENV infection, as well as in JEV and WNV infections (Su et al., 2002; Yu et al., 2006; Medigeshi et al., 2007; Pena et al., 2011). Activation of the IRE1 pathway typically leads to expression of both prosurvival and proapoptotic proteins, but in the case of DENV infection, IRE1-directed gene expression appears to favor prosurvival ER homeostasis and protein production. Expression of certain cellular proteins – such as the chaperone GRP78 that aids viral protein translocation to the ER lumen – assists the virus in ensuring continued viral protein translation and RNA replication in spite of cellular defenses (Wati et al., 2009). In addition to augmenting viral translation and replication, activation of the IRE1 pathway appears to play a regulatory role in inflammatory signaling during prolonged ER stress, promoting NF-κB activation when the UPR is initially induced but suppressing NF-κB function during prolonged ER stress (Kitamura, 2009). In this study, we have identified an additional mechanism by which DENV2 suppresses NF-κB signaling via the UPR. In WNV (Kunjin) infected cells, the UPR also attenuates the innate immune response through inhibition of type I interferon receptor signaling (Ambrose and Mackenzie, 2011). We have shown here that induction of SIAH1 via the UPR also reduces interferon signaling in addition to inflammatory cytokine expression. These works suggest that drugs targeting the UPR, already a focus of research as potential chemotherapeutics and treatments for inflammatory diseases, may have the potential to serve a therapeutic role in humans infected with DENV.

The ubiquitination and degradation of MyD88 we describe here is consistent with studies demonstrating the appropriation of the UPS by flaviviruses. Several key components of the UPS can be detected in the blood of dengue fever patients and have been found upregulated in a variety of cell lines during DENV infection (Fink et al., 2007; Gilfoy et al., 2008; Kanlaya et al., 2010; Fernandez-Garcia et al., 2011). Inhibition of the UPS strongly inhibits DENV and WNV RNA replication (Fink et al., 2007; Gilfoy et al., 2008; Kanlaya et al., 2010; Fernandez-Garcia et al., 2011). Notably, flavivirus infection appears to trigger proteasomal targeting and degradation of a variety of innate antiviral immune proteins: the most well-studied being the binding of DENV NS5 to STAT2, resulting in the ubiquitination and degradation of STAT2 and reduction in the type 1 interferon response (Ashour et al., 2009, 2010; Mazzon et al., 2009). JEV appears to abrogate antiviral innate immune signaling by triggering the degradation of viperin through an unknown mechanism (Chan et al., 2008). Although infection with JEV results in interferon-induced transcription of viperin, protein levels are reduced in a proteasome-dependent manner. Work by Zhu et al. demonstrated that cells infected with JEV express the miR-15/16 family member miR-15b, which in turn inhibits translation of the ubiquitin ligase RNF125, responsible for targeting RIG-I for proteasomal degradation (Zhu et al., 2015). Although miR-15b and miR-424 share the 6mer seed sequence characteristic of the miR-15/16 family, we did not observe an effect of miR-424 on RNF125 transcript expression (data not shown). Nonetheless, these studies suggest that reducing degradation of immune signaling molecules such as RIG-I and MyD88 may be a conserved function of the miR-15/16 family of miRNAs. Further study will be required to determine the breadth of control miR-15/16 family members exert on the proteasome-mediated degradation of immune signaling proteins.

## Data Availability Statement

All datasets generated for this study are included in the article/supplementary material.

## Author Contributions

AM, JS, and AH designed and performed the experiments, and analyzed the data. KP and VD provided additional reagents and protocols.

## Conflict of Interest

The authors declare that the research was conducted in the absence of any commercial or financial relationships that could be construed as a potential conflict of interest.

## References

[B1] Agarwal V. Bell G. W. Nam J.-W. Bartel D. P. (2015). Predicting effective microRNA target sites in mammalian mRNAs. *eLife* 4:101. 10.7554/eLife.05005 26267216 PMC4532895

[B2] Aguirre S. Maestre A. M. Pagni S. Patel J. R. Savage T. Gutman D. (2012). DENV inhibits type I IFN production in infected cells by cleaving human sting. *PLoS Pathog.* 8:e1002934. 10.1371/journal.ppat.1002934 23055924 PMC3464218

[B3] Akira S. Takeda K. (2004). Toll-like receptor signalling. *Nat. Rev. Immunol.* 4 499–511. 10.1038/nri1391 15229469

[B4] Ambrose R. L. Mackenzie J. M. (2011). West Nile virus differentially modulates the unfolded protein response to facilitate replication and immune evasion. *J. Virol.* 85 2723–2732. 10.1128/JVI.02050-10 21191014 PMC3067947

[B5] Ashour J. Laurent-Rolle M. Shi P. Y. Garcia-Sastre A. (2009). NS5 of Dengue virus mediates STAT2 binding and degradation. *J. Virol.* 83 5408–5418. 10.1128/JVI.02188-08 19279106 PMC2681973

[B6] Ashour J. Morrison J. Laurent-Rolle M. Belicha-Villanueva A. Plumlee C. R. Bernal-Rubio D. (2010). Mouse STAT2 restricts early Dengue virus replication. *Cell Host Microbe* 8 410–421. 10.1016/j.chom.2010.10.007 21075352 PMC3310429

[B7] Bhatt S. Gething P. W. Brady O. J. Messina J. P. Farlow A. W. Moyes C. L. (2013). The global distribution and burden of Dengue. *Nature* 496 504–507. 10.1038/nature12060 23563266 PMC3651993

[B8] Byk L. A. Iglesias N. G. De Maio F. A. Gebhard L. G. Rossi M. Gamarnik A. V. (2016). Dengue virus genome uncoating requires Ubiquitination. *mBio* 7:504. 10.1128/mBio.00804-16 27353759 PMC4937216

[B9] Chamorro-Jorganes A. Araldi E. Penalva L. O. F. Sandhu D. Fernández-Hernando C. Suárez Y. (2011). MicroRNA-16 and microRNA-424 regulate cell-autonomous angiogenic functions in endothelial cells via targeting vascular endothelial growth factor receptor-2 and fibroblast growth factor receptor-1. *Arterioscler. Thromb. Vasc. Biol.* 31 2595–2606. 10.1161/ATVBAHA.111.236521 21885851 PMC3226744

[B10] Chan Y.-L. Chang T.-H. Liao C.-L. Lin Y.-L. (2008). The cellular antiviral protein viperin is attenuated by proteasome-mediated protein degradation in Japanese encephalitis virus-infected cells. *J. Virol.* 82 10455–10464. 10.1128/JVI.00438-08 18768981 PMC2573197

[B11] Dallavalle C. Albino D. Civenni G. Merulla J. Ostano P. Mello-Grand M. (2016). MicroRNA-424 impairs Ubiquitination to activate STAT3 and promote prostate tumor progression. *J. Clin. Invest.* 126 4585–4602. 10.1172/JCI86505 27820701 PMC5127674

[B12] De Arras L. Seng A. Lackford B. Keikhaee M. R. Bowerman B. Freedman J. H. (2013). An evolutionarily conserved innate immunity protein interaction network. *J. Biol. Chem.* 288 1967–1978. 10.1074/jbc.M112.407205 23209288 PMC3548504

[B13] Diwaker D. Mishra K. P. Ganju L. (2015). Effect of modulation of unfolded protein response pathway on Dengue virus infection. *Acta Biochim. Biophys. Sin.* 47 960–968. 10.1093/abbs/gmv108 26515795

[B14] Fernandez-Garcia M. D. Meertens L. Bonazzi M. Cossart P. Arenzana-Seisdedos F. Amara A. (2011). Appraising the roles of CBLL1 and the Ubiquitin/proteasome system for flavivirus entry and replication. *J. Virol.* 85 2980–2989. 10.1128/JVI.02483-10 21191016 PMC3067953

[B15] Fink J. Gu F. Ling L. Tolfvenstam T. Olfat F. Chin K. C. (2007). Host gene expression profiling of Dengue virus infection in cell lines and patients. *PLoS Negl. Trop. Dis.* 1:e86. 10.1371/journal.pntd.0000086.g005 18060089 PMC2100376

[B16] Finnerty J. R. Wang W.-X. Hébert S. S. Wilfred B. R. Mao G. Nelson P. T. (2010). The miR-15/107 group of MicroRNA genes: evolutionary biology, cellular functions, and roles in human diseases. *J. Mol. Biol.* 402 491–509. 10.1016/j.jmb.2010.07.051 20678503 PMC2978331

[B17] Gilfoy F. Fayzulin R. Mason P. W. (2008). West Nile virus genome amplification requires the functional activities of the proteasome. *Virology* 385 74–84. 10.1016/j.virol.2008.11.034 19101004 PMC7103393

[B18] Green A. M. Beatty P. R. Hadjilaou A. Harris E. (2014). Innate immunity to Dengue virus infection and subversion of antiviral responses. *J. Mol. Biol.* 426 1148–1160. 10.1016/j.jmb.2013.11.023 24316047 PMC4174300

[B19] Gupta A. Hossain M. M. Read D. E. Hetz C. Samali A. Gupta S. (2015). PERK regulated miR-424(322)-503 cluster fine-tunes activation of IRE1 and ATF6 during unfolded protein response. *Sci. Rep.* 5:18304. 10.1038/srep18304 26674075 PMC4682135

[B20] Halstead S. B. (2017). Achieving safe, effective, and durable Zika virus vaccines: lessons from Dengue. *Lancet Infect. Dis.* 17 e378–e382. 10.1016/S1473-3099(17)30362-6 28711586

[B21] Hemmi H. Kaisho T. Takeuchi O. Sato S. Sanjo H. Hoshino K. (2002). Small anti-viral compounds activate immune cells via the TLR7 MyD88-dependent signaling pathway. *Nat. Immunol.* 3 196–200. 10.1038/ni758 11812998

[B22] Imig J. Motsch N. Zhu J. Y. Barth S. Okoniewski M. Reineke T. (2011). microRNA profiling in Epstein-Barr virus-associated B-cell lymphoma. *Nucleic Acids Res.* 39 1880–1893. 10.1093/nar/gkq1043 21062812 PMC3061055

[B23] Jiang X.-P. Ai W.-B. Wan L.-Y. Zhang Y.-Q. Wu J.-F. (2017). The roles of microRNA families in hepatic fibrosis. *Cell Biosci.* 7:34. 10.1186/s13578-017-0161-7 28680559 PMC5496266

[B24] Kanlaya R. Pattanakitsakul S.-N. Sinchaikul S. Chen S.-T. Thongboonkerd V. (2010). The Ubiquitin-proteasome pathway is important for Dengue virus infection in primary human endothelial cells. *J. Proteome Res.* 9 4960–4971. 10.1021/pr100219y 20718508

[B25] Kitamura M. (2009). Biphasic, bidirectional regulation of NF-kappaB by endoplasmic reticulum stress. *Antioxid. Redox Signal.* 11 2353–2364. 10.1089/ars.2008.2391 19187000

[B26] Leung A. K. L. Sharp P. A. (2010). MicroRNA functions in stress responses. *Mol. Cell.* 40 205–215. 10.1016/j.molcel.2010.09.027 20965416 PMC2996264

[B27] Lin Y. L. Liu C. C. Lei H. Y. Yeh T. M. Lin Y. S. Chen R. M. (2000). Infection of five human liver cell lines by Dengue-2 virus. *J. Med. Virol.* 60 425–431. 10.1002/(sici)1096-9071(200004)60:4<425::aid-jmv10>3.0.co;2-a 10686026

[B28] Loo Y.-M. Fornek J. Crochet N. Bajwa G. Perwitasari O. Martínez-Sobrido L. (2008). Distinct RIG-I and MDA5 signaling by RNA viruses in innate immunity. *J. Virol.* 82 335–345. 10.1128/JVI.01080-07 17942531 PMC2224404

[B29] Mazzon M. Jones M. Davidson A. Chain B. Jacobs M. (2009). Dengue virus NS5 inhibits interferon-alpha signaling by blocking signal transducer and activator of transcription 2 phosphorylation. *J. Infect. Dis.* 200 1261–1270. 10.1086/605847 19754307

[B30] Medigeshi G. R. Hirsch A. J. Brien J. D. Uhrlaub J. L. Mason P. W. Wiley C. (2009). West nile virus capsid degradation of claudin proteins disrupts epithelial barrier function. *J. Virol.* 83 6125–6134. 10.1128/JVI.02617-08 19369347 PMC2687390

[B31] Medigeshi G. R. Lancaster A. M. Hirsch A. J. Briese T. Lipkin W. I. Defilippis V. (2007). West Nile virus infection activates the unfolded protein response, leading to CHOP induction and apoptosis. *J. Virol.* 81 10849–10860. 10.1128/JVI.01151-07 17686866 PMC2045561

[B32] Meunier J. Lemoine F. Soumillon M. Liechti A. Weier M. Guschanski K. (2013). Birth and expression evolution of mammalian microRNA genes. *Genome Res.* 23 34–45. 10.1101/gr.140269.112 23034410 PMC3530682

[B33] Morrison J. Laurent-Rolle M. Maestre A. M. Rajsbaum R. Pisanelli G. Simon V. (2013). Dengue virus co-opts UBR4 to degrade STAT2 and antagonize type I interferon signaling. *PLoS Pathog.* 9:e1003265. 10.1371/journal.ppat.1003265 23555265 PMC3610674

[B34] Muñoz-Jordán J. L. Laurent-Rolle M. Ashour J. Martínez-Sobrido L. Ashok M. Lipkin W. I. (2005). Inhibition of alpha/beta interferon signaling by the NS4B protein of flaviviruses. *J. Virol.* 79 8004–8013. 10.1128/JVI.79.13.8004-8013.2005 15956546 PMC1143737

[B35] Padilla-S L. Rodríguez A. Gonzales M. M. Gallego-G J. C. Castaño-O J. C. (2014). Inhibitory effects of curcumin on Dengue virus type 2-infected cells in vitro. *Arch. Virol.* 159 573–579. 10.1007/s00705-013-1849-6 24081825

[B36] Pena J. Harris E. (2012). Early Dengue virus protein synthesis induces extensive rearrangement of the endoplasmic reticulum independent of the UPR and SREBP-2 pathway. *PLoS One* 7:e38202. 10.1371/journal.pone.0038202.g007 22675522 PMC3366941

[B37] Pena J. Pena J. Harris E. (2011). Dengue virus modulates the unfolded protein response in a time-dependent manner. *J. Biol. Chem.* 286 14226–14236. 10.1074/jbc.M111.222703 21385877 PMC3077624

[B38] Pryke K. M. Abraham J. Sali T. M. Gall B. J. Archer I. Liu A. (2017). A novel agonist of the TRIF pathway induces a cellular state refractory to replication of Zika, Chikungunya, and Dengue viruses. *mBio* 8:e452-17. 10.1128/mBio.00452-17 28465426 PMC5414005

[B39] Rodriguez-Madoz J. R. Belicha-Villanueva A. Bernal-Rubio D. Ashour J. Ayllon J. Fernandez-Sesma A. (2010). Inhibition of the type I interferon response in human dendritic cells by Dengue virus infection requires a catalytically active NS2B3 complex. *J. Virol.* 84 9760–9774. 10.1128/JVI.01051-10 20660196 PMC2937777

[B40] Sali T. M. Pryke K. M. Abraham J. Liu A. Archer I. Broeckel R. (2015). Characterization of a novel human-specific STING agonist that elicits antiviral activity against emerging alphaviruses. *PLoS Pathog.* 11:e1005324. 10.1371/journal.ppat.1005324 26646986 PMC4672893

[B41] Sanjana N. E. Shalem O. Zhang F. (2014). Improved vectors and genome-wide libraries for CRISPR screening. *Nat. Methods* 11 783–784. 10.1038/nmeth.3047 25075903 PMC4486245

[B42] Santhakumar D. Forster T. Laqtom N. N. Fragkoudis R. Dickinson P. Abreu-Goodger C. (2010). Combined agonist-antagonist genome-wide functional screening identifies broadly active antiviral microRNAs. *Proc. Natl. Acad. Sci. U.S.A.* 107 13830–13835. 10.1073/pnas.1008861107 20643939 PMC2922253

[B43] Sariol C. A. Martinez M. I. Rivera F. Rodríguez I. V. Pantoja P. Abel K. (2011). Decreased Dengue replication and an increased anti-viral humoral response with the use of combined Toll-like receptor 3 and 7/8 agonists in macaques. *PLoS One* 6:e19323. 10.1371/journal.pone.0019323 21559444 PMC3084804

[B44] Schmittgen T. D. Livak K. J. (2008). Analyzing real-time PCR data by the comparative C(T) method. *Nat. Protoc.* 3 1101–1108. 10.1038/nprot.2008.73 18546601

[B45] Scortegagna M. Kim H. Li J.-L. Yao H. Brill L. M. Han J. (2014). Fine tuning of the UPR by the Ubiquitin ligases Siah1/2. *PLoS Genet.* 10:e1004348. 10.1371/journal.pgen.1004348 24809345 PMC4014425

[B46] Smith J. L. Grey F. E. Uhrlaub J. L. Nikolich-Zugich J. Hirsch A. J. (2012). Induction of the cellular microRNA, Hs_154, by West Nile virus contributes to virus-mediated apoptosis through repression of antiapoptotic factors. *J. Virol.* 86 5278–5287. 10.1128/JVI.06883-11 22345437 PMC3347395

[B47] Smith J. L. Jeng S. McWeeney S. K. Hirsch A. J. (2017). A MicroRNA screen identifies the Wnt signaling pathway as a regulator of the interferon response during flavivirus infection. *J. Virol.* 91:e2388-16. 10.1128/JVI.02388-16 28148804 PMC5375670

[B48] Stanaway J. D. Shepard D. S. Undurraga E. A. Halasa Y. A. Coffeng L. E. Brady O. J. (2016). The global burden of Dengue: an analysis from the Global Burden of Disease Study 2013. *Lancet Infect. Dis.* 16 712–723. 10.1016/S1473-3099(16)00026-8 26874619 PMC5012511

[B49] Stein D. A. Perry S. T. Buck M. D. Oehmen C. S. Fischer M. A. Poore E. (2011). Inhibition of Dengue virus infections in cell cultures and in AG129 mice by a small interfering RNA targeting a highly conserved sequence. *J. Virol.* 85 10154–10166. 10.1128/JVI.05298-11 21795337 PMC3196423

[B50] Su H.-L. Liao C.-L. Lin Y.-L. (2002). Japanese encephalitis virus infection initiates endoplasmic reticulum stress and an unfolded protein response. *J. Virol.* 76 4162–4171. 10.1128/jvi.76.9.4162-4171.2002 11932381 PMC155064

[B51] Tsai Y.-T. Chang S.-Y. Lee C.-N. Kao C.-L. (2009). Human TLR3 recognizes Dengue virus and modulates viral replication in vitro. *Cell Microbiol.* 11 604–615. 10.1111/j.1462-5822.2008.01277.x 19134117

[B52] Wang J. Huo K. Ma L. Tang L. Li D. Huang X. (2011). Toward an understanding of the protein interaction network of the human liver. *Mol. Syst. Biol.* 7:536. 10.1038/msb.2011.67 21988832 PMC3261708

[B53] Wati S. Soo M. L. Zilm P. Li P. Paton A. W. Burrell C. J. (2009). Dengue virus infection induces upregulation of GRP78, which acts to chaperone viral antigen production. *J. Virol.* 83 12871–12880. 10.1128/JVI.01419-09 19793816 PMC2786853

[B54] Welsch S. Miller S. Romero-Brey I. Merz A. Bleck C. K. E. Walther P. (2009). Composition and three-dimensional architecture of the Dengue virus replication and assembly sites. *Cell Host Microbe* 5 365–375. 10.1016/j.chom.2009.03.007 19380115 PMC7103389

[B55] Wu K. Hu G. He X. Zhou P. Li J. He B. (2013). MicroRNA-424-5p suppresses the expression of SOCS6 in pancreatic cancer. *Pathol. Oncol. Res.* 19 739–748. 10.1007/s12253-013-9637-x 23653113

[B56] Xiao S. Y. Guzman H. Zhang H. Travassos da Rosa A. P. Tesh R. B. (2001). West Nile virus infection in the golden hamster (Mesocricetus auratus): a model for West Nile encephalitis. *Emerg. Infect. Dis.* 7 714–721. 10.3201/eid0704.017420 11585537 PMC2631753

[B57] Xiao X. Huang C. Zhao C. Gou X. Senavirathna L. K. Hinsdale M. (2015). Regulation of myofibroblast differentiation by miR-424 during epithelial-to-mesenchymal transition. *Arch. Biochem. Biophys.* 566 49–57. 10.1016/j.abb.2014.12.007 25524739 PMC4297572

[B58] Yu C.-Y. Hsu Y.-W. Liao C.-L. Lin Y.-L. (2006). Flavivirus infection activates the XBP1 pathway of the unfolded protein response to cope with endoplasmic reticulum stress. *J. Virol.* 80 11868–11880. 10.1128/JVI.00879-06 16987981 PMC1642612

[B59] Zhu B. Ye J. Nie Y. Ashraf U. Zohaib A. Duan X. (2015). MicroRNA-15b modulates Japanese encephalitis virus-mediated inflammation via targeting RNF125. *J. Immunol.* 195 2251–2262. 10.4049/jimmunol.1500370 26202983

